# Characterization of two transcriptomic subtypes of marker-null large cell carcinoma of the lung suggests different origin and potential new therapeutic perspectives

**DOI:** 10.1007/s00428-023-03721-4

**Published:** 2024-01-03

**Authors:** Michele Simbolo, Giovanni Centonze, Anastasios Gkountakos, Valentina Monti, Patrick Maisonneuve, Stela Golovco, Giovanna Sabella, Alessandro Del Gobbo, Stefano Gobbo, Stefano Ferrero, Alessandra Fabbri, Carlotta Pardo, Giovanna Garzone, Natalie Prinzi, Sara Pusceddu, Adele Testi, Luigi Rolli, Alessandro Mangogna, Luisa Bercich, Mauro Roberto Benvenuti, Emilio Bria, Sara Pilotto, Alfredo Berruti, Ugo Pastorino, Carlo Capella, Maurizio Infante, Michele Milella, Aldo Scarpa, Massimo Milione

**Affiliations:** 1 Section of Pathology, Department of Diagnostics and Public Health, University of Verona, Verona, Italy; 2 1st Pathology Division, Department of Pathology and Laboratory Medicine, Fondazione IRCCS Istituto Nazionale Dei Tumori, Milan, Italy; 3 Division of Epidemiology and Biostatistics, IEO, European Institute of Oncology IRCCS, Milan, Italy; 4 Division of Pathology, Fondazione IRCCS Ca’ Granda Ospedale Maggiore Policlinico, Milan, Italy; 5 Department of Traslational Medicine, University of Ferrara, Ferrara, Italy; 6 2nd Pathology Division, Department of Pathology and Laboratory Medicine, Fondazione IRCCS Istituto Nazionale Dei Tumori, Milan, Italy; 7 Medical Oncology Department, Fondazione IRCCS, Istituto Nazionale Dei Tumori, Milan, Italy; 8 Thoracic Surgery Unit, Fondazione IRCCS Istituto Nazionale Tumori, Milan, Italy; 9 Institute for Maternal and Child Health, IRCCS Burlo Garofalo, Trieste, Italy; 10 Department of Pathology, ASST Spedali Civili of Brescia, Brescia, Italy; 11 Thoracic Surgery Unit, Department of Medical and Surgical Specialties Radiological Sciences and Public Health, Medical Oncology, University of Brescia, ASST Spedali Civili of Brescia, Brescia, Italy; 12 Fondazione Policlinico Universitario A. Gemelli IRCCS, Rome, Italy; 13 Section of Oncology, Department of Medicine, University of Verona, Verona, Italy; 14 Medical Oncology Unit, ASST Spedali Civili of Brescia, Department of Medical and Surgical Specialties, Radiological Science and Public Health, University of Brescia, Brescia, Italy; 15 Department of Medicine and Surgery, University of Insubria, Varese, Italy; 16 Thoracic Surgery, Hospital Trust of Verona, Verona, Italy; 17 ARC-NET Research Centre for Applied Research On Cancer, University and Hospital Trust of Verona, Piazzale Scuro, 10, 37134 Verona (VR), Italy

**Keywords:** Lung, Large cell carcinoma, Next generation sequencing, Transcriptomics, Biomarkers, Aim2, Pou2f3

## Abstract

Pulmonary large cell carcinoma (LCC) is an undifferentiated neoplasm lacking morphological, histochemical, and immunohistochemical features of small cell lung cancer, adenocarcinoma (ADC), or squamous cell carcinoma (SCC). The available molecular information on this rare disease is limited. This study aimed to provide an integrated molecular overview of 16 cases evaluating the mutational asset of 409 genes and the transcriptomic profiles of 20,815 genes. Our data showed that *TP53* was the most frequently inactivated gene (15/16; 93.7%) followed by *RB1* (5/16; 31.3%) and *KEAP1* (4/16; 25%), while *CRKL* and *MYB* genes were each amplified in 4/16 (25%) cases and *MYC* in 3/16 (18.8%) cases; transcriptomic analysis identified two molecular subtypes including a Pure-LCC and an adenocarcinoma like-LCC (ADLike-LCC) characterized by different activated pathways and cell of origin. In the Pure-LCC group, *POU2F3* and *FOXI1* were distinctive overexpressed markers. A tuft cell-like profile and the enrichment of a replication stress signature, particularly involving ATR, was related to this profile. Differently, the ADLike-LCC were characterized by an alveolar-cell transcriptomic profile and association with *AIM2* inflammasome complex signature. In conclusion, our study split the histological marker-null LCC into two different transcriptomic entities, with *POU2F3*, *FOXI1*, and *AIM2* genes as differential expression markers that might be probed by immunohistochemistry for the differential diagnosis between Pure-LCC and ADLike-LCC. Finally, the identification of several signatures linked to replication stress in Pure-LCC and inflammasome complex in ADLike-LCC could be useful for designing new potential therapeutic approaches for these subtypes.

**Supplementary Information:**

The online version contains supplementary material available at 10.1007/s00428-023-03721-4.

## Introduction

In the last decade, the combination of pathologic, genomic, and clinical advances has led to reclassification of large cell carcinomas (LCC) of the lung into more specific pathologic entities [16]. Indeed, the 2021 World Health Organization (WHO) classification defines pulmonary LCC as a rare undifferentiated carcinoma that lacks the cytological, architectural, immunohistochemical, and histochemical features of small cell lung cancer, adenocarcinoma (ADC), or squamous cell carcinoma (SCC) [17]. In detail, if a lung cancer with large cell morphology expresses immunohistochemical markers of pneumocytes, such as thyroid transcription factor 1 (TTF-1) and NapsinA, it is considered ADC. Conversely, if squamous markers including p40, CK5/6, or p63 are expressed, the lung cancer is defined as SCC. Additionally, if it is positive for the neuroendocrine markers synaptophysin and chromogranin, it is considered large cell neuroendocrine carcinoma (LCNEC). Therefore, LCC is a diagnosis of exclusion in a surgically resected NSCC lacking expression of the aforementioned immunohistochemical markers and mucin stains [1, 17].

Identification of molecular drivers and potential therapeutic targets in LCC would result in a clinically meaningful adjustment in disease management. However, the molecular characterization of these tumours remains challenging due to their rarity. The available information on genomic alterations consists of three studies performed using different targeted next generation sequencing gene panels on 12 (26 genes analysed) [4], 25 (166 genes analysed) [1], and 7 (425 genes analysed) [8] cases, which agree on *TP53* as the most frequently mutated gene. Furthermore, only one gene expression analysis was carried out on 12 cases, suggesting the presence of two molecular profiles, one of which was linked to mitogenic processes and the second was similar to that of ADC [5].

The present study aimed to gather further information on this rare disease entity by providing an integrated molecular overview of 16 cases of LCC based on the evaluation of the mutational asset of 409 genes and the transcriptomic profiles of 20,815 genes.

## Materials and methods

### Cases

The clinical databases of three Italian hospitals (Fondazione IRCCS Istituto Nazionale dei Tumori, Milan; ASST Spedali Civili di Brescia, Brescia; Fondazione IRCCS Ca’ Granda Ospedale Maggiore Policlinico, Milan), between 2010 and 2020, were queried for the diagnosis “large cell carcinoma”. Twenty-eight cases were identified and revised by six pathologists (C.C., M.M., A.S., A.F., L.B., G.S.). Twelve cases were excluded: three because only bioptic or cytologic material was available; 9 were excluded after immunostaining: 6 positive for TTF1 and NapsinA were defined as ADC with solid pattern; 2 positive for p40 were defined as non-keratinizing SCC; and one case immunoexpressed chromogranin A and synaptophysin and was defined LCNEC. Finally, 16 cases met all the LCC criteria of the WHO 2021 classification [17] Table 1); none of these 16 cases showed any Alcian or PAS histochemical stain.

**Table 1:** Clinicopathological features of 16 large cell carcinomas (LCCs)

ID	Age	Gender	Smoke	Mitosis (*n*)	Necrosis	pT	pN	pM	Stage	FU (months)	Vital status	Cluster
2	71	Female	Current	36	extended	2	0	0	I	95	DOD	LCC
3	65	Male	Current	23	extended	2	0	0	I	2	DOD	ADLike
8	83	Male	Current	44	spotted	1	0	0	I	17	DOD	LCC
13	66	Male	Current	17	extended	1	1	1	IV	20	DOD	LCC
15	60	Male	Former	14	absent	2	0	0	II	45	AWD	ADLike
21	57	Male	Current	14	absent	1	0	0	I	17	AWD	ADLike
22	68	Male	Former	9	extended	1	2	0	III	13	AWD	ADLike
84	51	Male	Current	19	extended	1	0	0	I	242	AWD	LCC
87	66	Male	Current	39	absent	1	1	0	II	10	DOD	LCC
182	80	Male	Former	26	extended	2	0	0	I	48	DOD	LCC
294	70	Male	Current	42	extended	3	1	0	III	18	AWD	LCC
348	74	Male	Current	30	spotted	2	0	0	I	36	DOD	LCC
350	74	Male	Never	19	extended	2	0	0	I	24	AWD	LCC
494	77	Male	Former	43	extended	2	0	0	I	7	DOD	LCC
VAL16	84	Male	Current	20	extended	1	0	0	I	15	DOD	LCC
VAL26	60	Male	Former	11	spotted	2	0	0	II	25	AWD	ADLike

*FU*, follow up; *DOD*, dead of disease; *AWD*, alive without disease; cluster: according to transcription profile

In addition, 17 ADC and 11 LCNEC cases were used for a comparative transcriptomic profiling.

The study was performed according to the clinical standards of the 1983 Declaration of Helsinki and was approved by the Ethic Committee of Fondazione IRCCS INT (No. INT 171/16).

### Immunohistochemistry

Immunostaining was performed for the 10 markers listed in Table 2 in an automated immunostainer (Dako Autostainer System). The antibodies Pou Class 2 homeobox 3 (Pou2f3), absent in melanoma 2 (Aim2), and forkhead box I1 (Foxi1) were tested to validate transcriptomic findings and were evaluated as a percentage of positive cells according to Yamada et al*. *[24].

**Table 2:** Antibody sources and dilutions

Antigen	Pretreatment	Dilution	Code Number	Clone	Source
NapsinA (M)	High pH 30 min — 96 °C	1/500	NCL-L-Napsin A	IP64	Leica Biosystems
TTF-1 (M)	High pH 30 min — 96 °C	1/2000	M3575	8G7G3	Dako, Agilent
p40 (M)	High pH 60 min — 96 °C	1/400	API 3079 G3	BC28	Biocare Medical
Chromogranin-A (M)	High pH 60 min — 98 °C	1/100	M0869	Dak-A3	Dako, Agilent
Synaptophisin (M)	High pH 15 min — 96 °C	1/200	M7315	Dak-Synap	Dako, Agilent
Smarca4 (M)	High pH 40 min — 96 °C	1/100	sc-17796	G-7	Santa Cruz
PDL1 (M)	High pH 15 min — 96 °C	1/50	SK006	22c3	Dako, Agilent
Pou2f3 (P)	Low pH 15 min — 96 °C	1/200	HPA019652	Polyclonal	Sigma-Aldrich
Aim2 (P)	Low pH 15 min — 96 °C	1/200	HPA031365	Polyclonal	Sigma-Aldrich
Foxi1 (P)	High pH 30 min — 96 °C	1/500	HPA071469	Polyclonal	Sigma-Aldrich

*M*, monoclonal; *P*, Polyclonal; *Ki-67*, Ki67 index; *TTF-1*, thyroid transcription factor 1; *Pou2f3*, POU class 2 homeobox 3; *Gli1*, glioma-associated oncogene family zinc finger 1; *Yap1*, Yes1 associated transcriptional regulator; *Aim2*, absent in melanoma 2; *Foxi1*, forkhead box I1

### Mutational and copy number variation status of 409 cancer genes

DNA was obtained from FFPE tumour using 10 consecutive 4-μm sections and the QIAamp DNA FFPE Tissue Kit (Qiagen, Milan, Italy). DNA was qualified as reported elsewhere [19]. The Oncomine Tumour Mutational Load (TML) panel (Thermo Fisher Scientific, Milan, Italy) with next-generation sequencing assay was used. The assay covers 1.65 Mb including the exons of 409 cancer-related genes (Supplementary Methods).

### Tumour mutational load and mutational signatures

Tumour mutational load (TML) and mutational spectrum for each sample were evaluated using the Oncomine TML 5.10 plugin on IonReporter (Thermo Fisher Scientific) as detailed in Supplementary Methods.

### FISH validation of MYB gene amplification

FISH assay was carried out to assess *MYB* (6q23.3) amplification using a Locus-Specific Probes XL 6q21/6q23/6cen (MetaSystems srl Italia). An orange fluorochrome labelled to hybridize the *MYB* gene localized on 6q23 and an aqua fluorochrome labelled to hybridize the centromere.

### Fusion genes and splice variant detection

*ALK*, *RET*, and *ROS1* rearrangements and *MET* exon skipping were investigated using an automated real time polymerase chain reaction (RT-PCR) approach (Easy PGX platform, Diatech Pharmacogenetics, Jesi, Italy).

### Expression analysis by next-generation sequencing

RNA was prepared using ReliaPrep FFPE Total RNA Miniprep System (Promega, Milan, Italy), quantified using Qubit RNA HS Assay Kit (Thermo Fisher), and qualified using RIN analysis of Agilent RNA 6000 Nano Kit on Agilent 2100 Bioanalyzer (Agilent Technologies). RNA with RIN > 5 and concentration over 10 ng/µl was considered suitable. The Ampliseq Transcriptome Human Gene Expression Kit (Thermo Fisher Scientific, MA, USA) was used to analyse the expression status of 20,815 human RefSeq genes (Supplementary Methods). The expression data analysis was subjected to quality control using the workflow defined by Law et al*.* [6].

### Gene set enrichment analysis (GSEA)

To identify the biological processes differently enriched among all the clusters, we used GAGE R package [10] and ssGSEA score [20]. We identified the cluster-specific enriched gene sets using pathways from MSigDB [9, 20]. We assessed the ssGSEA score and performed a *z*-score normalization of the pathway for each sample (Supplementary Methods). A positive correlation between the sample and the specific pathway is represented by a *z*-score > 0. We considered only the differently related pathways (*p*-value < 0.05 according to Benjamini–Hochberg test). All samples were grouped according to their molecular class.

### Statistical analysis

The association between immunophenotypical and molecular features and their correlation with different LCC groups (ADLike-LCC vs. Pure-LCC) was assessed using the Fisher exact test for categorical variables and the nonparametric Wilcoxon test for continuous variables. Data analysis was performed using MedCalc Software. All tests were two-sided and *p*-values < 0.05 were considered statistically significant.

## Results

### Clinicopathological features

The clinicopathological features of the 16 marker-null LCC are summarized in Table 1. The series comprised 15 (94%) males and 1 (6%) female with a median age of 69 years (range: 51–84 years). All except one were current smokers (10/16; 62.5%) or had a history of smoking (5/16; 31.3%). Follow-up was available for all patients (mean 41 months); three cases (18.7%) were metastatic at diagnosis, and 9 patients (56%) died of the disease.

The morphological findings were characterized by solid neoplastic tissue composed of large polygonal cells with prominent nucleoli; necrosis was present in 13 cases (81.3%).

### Mutational and copy number status of 409 genes

Genomic sequencing achieved an average coverage of 346 × (122–768 ×) in tumour and 281 × (120–546) in matched normal samples (Supplementary Table 1).

**Fig. 1:**
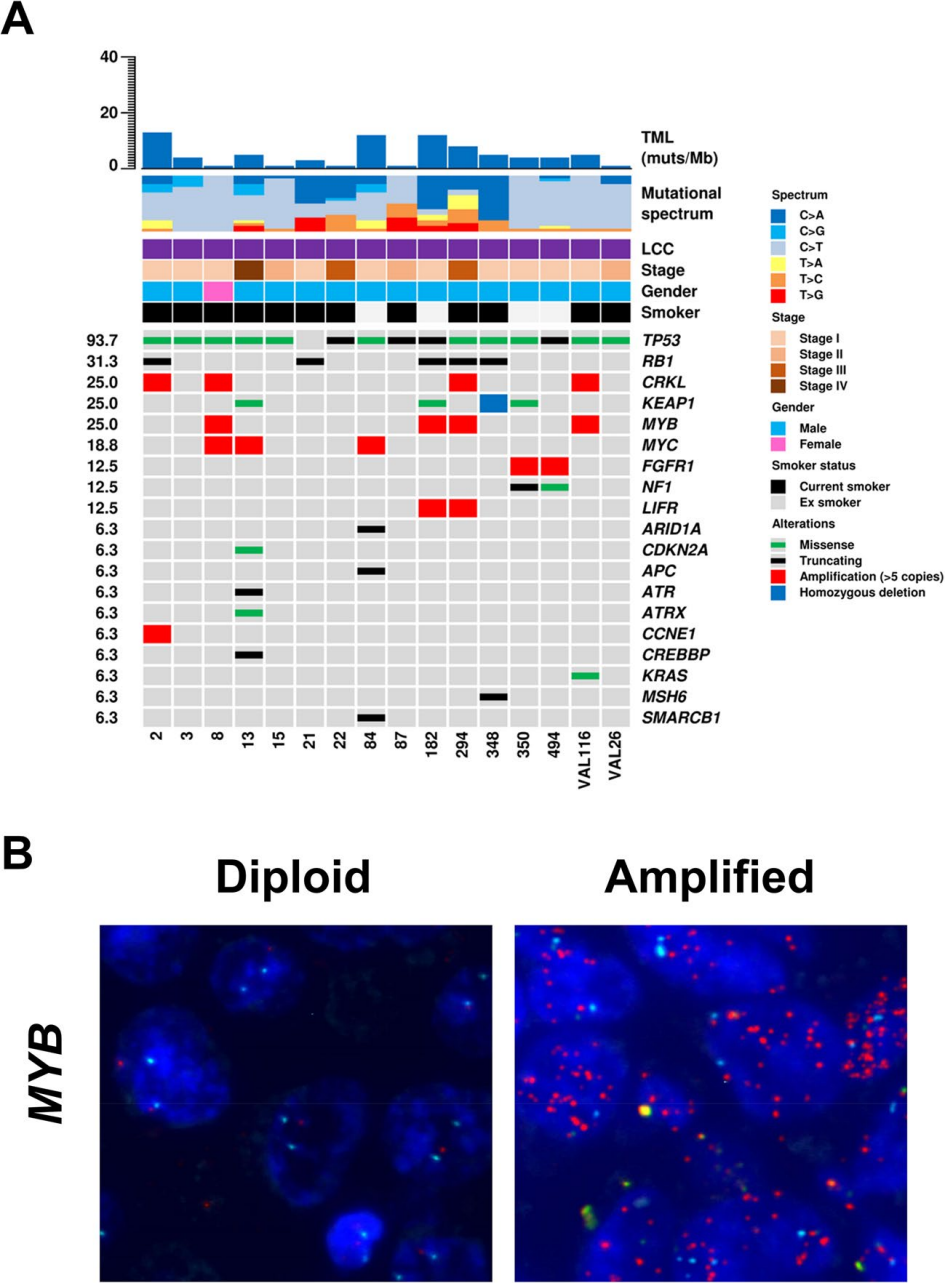
Genomic features of LCC and FISH validation for *MYB* gene amplification. **A** The upper histogram shows the tumour mutational load, defined as the number of mutations per megabase (muts/Mb), of each sample. The central matrix shows 19 genes that were found altered at sequencing analysis. Genes are listed according to the frequency of alterations. **B** Representative images of the FISH validation for *MYB* gene of a diploid (on the left) and an amplified case (on the right). Red spots mark *MYB* gene, while the spectrum green spots label the centromere of chromosome 6

Mutations were found in at least one gene in all 16 cases (Fig. 1A, Supplementary Table 2). A total of 35 mutations in 14 genes were identified, including 20 missense, 4 nonsense, 5 splice site alterations, 1 small deletion, and 5 frameshift (Supplementary Table 2).

The most frequent mutations involved *TP53* (15/16; 93.7%), followed by *RB1* (5/16; 31.3%) and *KEAP1* (4/16; 25.0%). One case had a *KRAS* A146T mutation [14]. Two cases harboured *EGFR* non-canonical mutations: a deletion of 21 nucleotides of exon 1 and an E884K missense mutation in exon 22 [18].

TML value, molecular spectrum, and COSMIC signature were computed for each (Supplementary Table 3). A median of 4.4 mutations per Mb (range 0.8–12.7) was estimated for all LCCs, similar to that of lung adenocarcinomas [7]. The mutational signatures did not show specific patterns.

The CNV status was estimated for all 409 genes using sequencing data. Focal amplification was observed in 6 genes (Fig. 1A) including the most frequent: *MYB*, *CRKL* (each 4/16; 25.0%), and *MYC* (3/16; 18.8%). One sample showed homozygous deletion of *KEAP1* gene. The FISH validation for *MYB* gene confirmed the gene amplification in all 4 cases (Fig. 1B).

Based on the chromosomal position of each gene, the status of chromosome arms was inferred (Supplementary Fig. 1). The major alterations were gains in chromosomes 3, 5, 6, 8, and 20, while losses were observed in chromosomes 3, 5, 13, and 15.

### Fusion genes and splice variants

No fusion genes or splice variants were detected for *ALK*, *RET*, *ROS*, and *MET* genes.

### Comparison of marker-null LCC expression profiles with lung adenocarcinomas and large cell neuroendocrine cancers

We investigated the transcriptomic relationship between marker-null LCC, ADC, and LCNEC, which represent the other non-keratinizing large cell histotypes of lung cancer. An unsupervised clustering analysis was conducted for 16 LCC, 17 ADC, and 11 LCNEC samples using the highest variable expressed genes (HVGs; explaining 70% of the total variance) which resulted in 2109 genes. Consensus clustering [22] was applied to identify the best number of clusters (k) which resulted to be *k* = 3 (Supplementary Fig. 2).

**Fig. 2:**
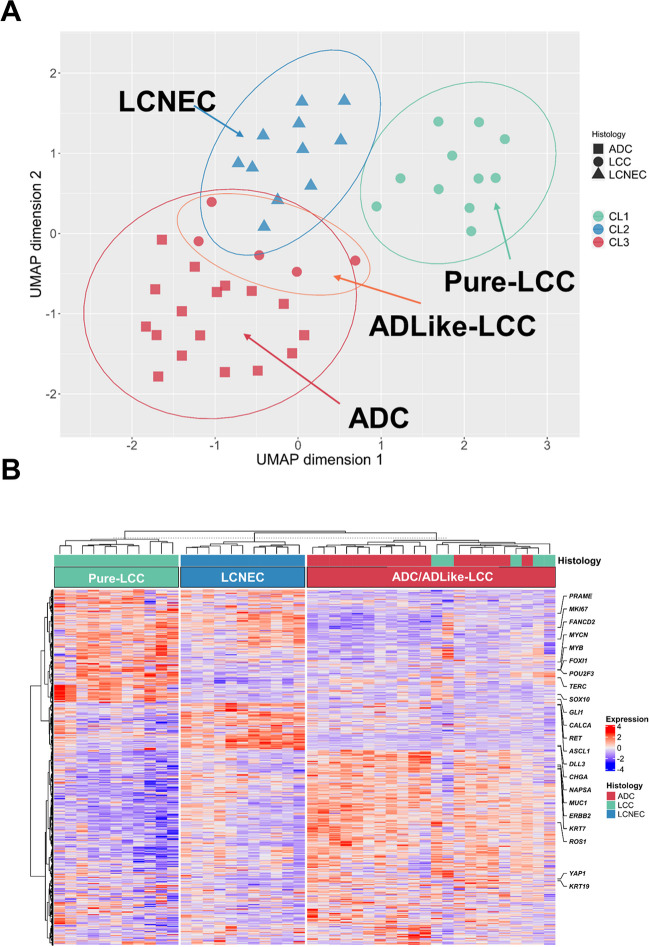
Gene expression analysis of LCC, ADC and LCNEC. Transcriptome sequencing data of 16 marker-null LCC, 17 ADC and 11 LCNEC are represented using two approaches: **A** Uniform manifold approximation and projection (UMAP) method using the highest variable expressed genes (HVGs; explaining 70% of the total variance), which were 2109 genes. ADC, adenocarcinoma; LCNEC, large cell neuroendocrine carcinoma; Pure-LCC, pure large cell carcinoma; ADLike-LCC, adenocarcinoma like large cell carcinoma. **B** Heatmap resulting from hierarchical clustering analysis using the 2109 HVGs, in which tumor samples are arranged in columns, grouped according to their expression clustering class, annotated for the histological subtype. The expression values of 2109 genes are indicated in red and blue to indicate high and low expression, respectively

An expression-based molecular map was developed using UMAP method to understand the topological relationships between samples [11]. Specifically, 11 marker-null LCC samples formed a standalone group (named Pure-LCC), while the remaining 5 cases were included in the cluster enriched for ADC histology and were named adenocarcinoma-like (ADLike-LCC; Fig. 2A). To understand the relationship between each sample and the others, we applied hierarchical clustering analysis that grouped the samples as follows (Fig. 2B): cluster 1 (CL1; Pure-LCC), including 11 marker-null LCC samples; cluster 2 (CL2; named LCNEC), including 11 LCNEC samples; and cluster 3 (CL3; named ADC/ADLike-LCC), including the remaining 22 samples, composed of 17 ADCs, and 5 marker-null LCCs (ADLike-LCC). The main clinicopathological characteristics of patients according to their expression profile are summarized in Table 3.

**Table 3:** Clinicopathological characteristics of 16 marker-null LCC according to their expression profile

	All Patients	Pure-LCC profile	ADLike-LCC profile	*p*-value*
Total	16 (100)	11 (100)	5 (100)	
Age				
Median [range]	69 [51–84]	74 [51–84]	60 [57–68]	**0.023**
Gender				
Female	1 (6.3)	1 (9.1)	0 (0.0)	
Male	15 (93.7)	10 (90.9)	5 (100.0)	1.00
Smoke				
Never	1 (6.3)	1 (9.1)	0 (0.0)	
Ex	5 (31.3)	2 (18.2)	3 (60.0)	
Current	10 (62.5)	8 (72.7)	2 (40.0)	0.35
Mitosis				
Median [range]	21.5 [9.0–44.0]	30.0 [17.0–44.0]	14.0 [9.0–23.0]	**0.009**
Necrosis				
Absent	3 (18.8)	1 (9.1)	2 (40.0)	
Spot	3 (18.8)	2 (18.2)	1 (20.0)	
Extensive	10 (62.5)	8 (72.7)	2 (40.0)	0.46
Stage				
I	10 (62.5)	8 (72.7)	2 (40.0)	
II	3 (18.8)	1 (9.1)	2 (40.0)	
III	2 (12.5)	1 (9.1)	1 (20.0)	
IV	1 (6.3)	1 (9.1)	0 (0.0)	0.43
Death event				
No	7 (43.8)	3 (27.3)	4 (80.0)	
Yes	9 (56.2)	8 (72.7)	1 (20.0)	0.11

*LCC*, large cell carcinoma; ADLike profile

** + **LCC with expression profiles similar to those of lung adenocarcinoma

*p-value based on Fisher’s exact for categorical variables and Wilcoxon test for continuous variables

Bold are indicated the statistically significant values

**Fig. 3:**
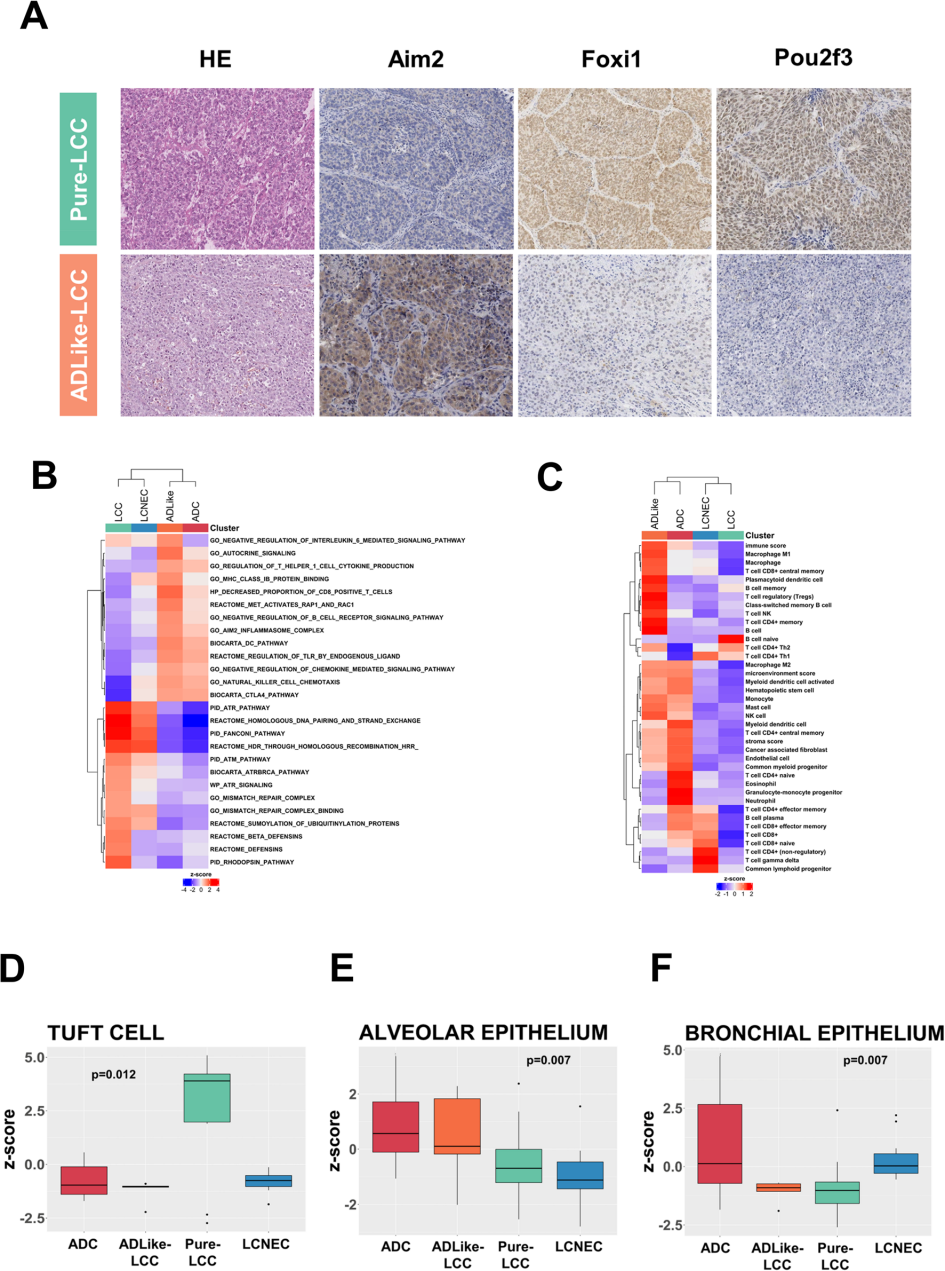
Immunohistochemical and gene set enrichment analysis (GSEA). **A** Differential immunostainings for Aim2, Foxi1 and Pou2f3 markers in pure large cell carcinoma (Pure-LCC) and in adenocarcinoma like LCC (ADLike-LCC) molecular subtypes. HE (haematoxylin and eosin). Heatmaps of **B** relevant gene sets from MSigDB collections; **C** immune subpopulations inferred by gene expression of immune metagenes significantly enriched in each of the four molecular classes (ADC, ADLike-LCC, Pure-LCC and LCNEC); **D**–**F** box and whisker plots displaying the normalized enrichment z-score for the tuft cell (**D**), alveolar (**E**) and bronchial (**F**) epithelium signatures. ssGSEA was used to obtain the enrichment score, representing the degree to which the genes in a particular gene set are co-ordinately up or downregulated

Differential expression (DE) analysis between clusters highlighted the overexpression of 121 LCC-specific genes. The *FOXI1* gene was the most representative overexpressed marker for the Pure-LCC group followed by *POU2F3, MYB*, and *KIT*, which showed the lowest adjusted *p*-value and the highest logFC (Supplementary Table 4). An immunostaining for the two most representative gene-related proteins, Foxi1 and Pou2f3 (Fig. 3A), was performed. Both markers resulted high expressed in the Pure-LCC compared to ADLike-LCC (*p* = 0.035 and *p* = 0.043, respectively) (Supplementary Fig. 3A, Supplementary Table 5).

Next, we performed gene set enrichment analysis (GSEA) to identify the main molecular pathways characterizing the Pure-LCC cluster. We observed a positive association with the biological process related to DNA repair through homologous recombination mechanisms, including Fanconi, *ATM* and *ATR* pathways (Fig. 3B). Alpha and beta defensin signalling was also enriched exclusively for this cluster together with cell proliferation and division processes. In fact, Pure-LCC showed a higher mitotic count compared to ADLike-LCC (*p* = 0.009, Table 3). Furthermore, a strong similarity to tuft cell profile described by Yamada et al*.* [24] was observed (Fig. 3D) due to overexpression of tuft cell markers as *FOXI1*, *GFI1B*, *HEPACAM2*, and *POU2F3* in this group.

Five of the 16 marker-null LCCs showed an ADLike-LCC expression profile. Although these cases scored negative at NapsinA immunostaining, they showed a transcriptomic profile characterized by overexpression of *NAPSA*, *FOS*, *Surfactant*, *S100A11*, and *YAP1* genes similar to that of ADC samples, whereas none of the 121 Pure-LCC specific genes was overexpressed. NapsinA immunostaining of *NAPSA* overexpressing cases highlighted that this protein was located in normal lung tissue within hyperplastic pneumocytes and macrophages (Supplementary Fig. 3B). The DE analysis identified 4 ADLike-LCC specific overexpressed genes: *AIM2*, *DKK1*, *S100A8*, and *SERPINB4*. Immunohistochemical analysis for Aim2 confirmed its expression in at least 60% of neoplastic cells of all five ADLike-LCC cases but only 3 of the 11 Pure-LCC samples (Supplementary Fig. 3A, Supplementary Table 1). ADLike-LCC cases were also distinguished from Pure-LCC by a low TML (median = 1.4 mut/Mb vs. 4.4 mut/Mb; *p* = 0.04). The GSEA highlighted the presence of a positive correlation among several pathways related to the inflammatory response, including the *AIM2* inflammasome complex but not *PDL1* (Supplementary Table 6). Of interest, immunostaining for PDL1 resulted negative in both ADLike-LCC and Pure-LCC. Then, we performed a deconvolution analysis comparing Pure-LCC with ADLike-LCC cases. As shown in Fig. 3D, this analysis revealed that the ADLike-LCC group was characterized by a strong infiltrate including macrophages, B lymphocytes, and dendritic cells. Finally, we investigated the cellular origin of ADLike-LCC using the 2 signatures described by Nakamura et al*.* [12] that comprises specific markers of lung alveolar and bronchial cells. According to the GSEA scoring, the ADLike-LCC showed an expression profile compatible with an alveolar origin (Fig. 3E) due to overexpression of several alveolar lung markers including *HIGD1B* and *RFTN*, and the lack of bronchial markers (Fig. 3F).

## Discussion

**Fig. 4:**
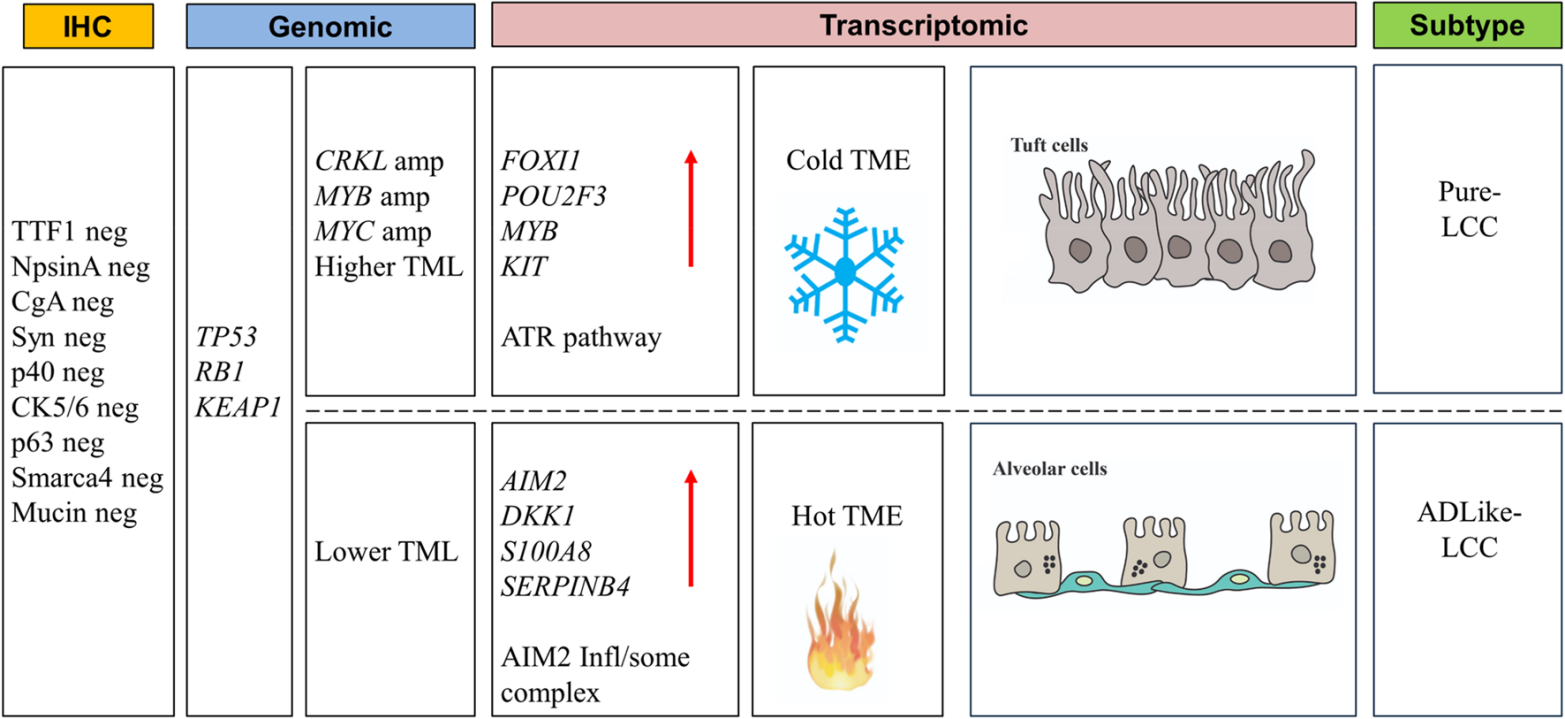
Schematic representation of the results of the study. Marker-null LCC were defined as cancers negative for immunohistochemical markers of lung adenocarcinoma (TTF-1, NapsinA), squamous cell carcinoma (p40, CK5/6, p63), and large cell neuroendocrine carcinoma (ChgA, Syn) and for mucin immunostaining (Alcian-PAS). Genomic analysis showed common (*TP53*, *RB1* and *KEAP1* mutations) and differential (amplification of *CRKL*, *MYB and MYC*; TML, tumor mutational load) alterations. Transcriptomic analysis identified two molecular subtypes: Pure-LCC and ADLike-LCC. These were characterized by different overexpressed genes (red arrows) and potentially targetable enriched pathways (ATR pathway and AIM2 inflammasome complex). Transcriptomes also revealed differences regarding the composition of tumour microenvironment (TME: cold and hot) and the cell of origin

The present study on the genomic and transcriptomic analysis of 16 marker-null LCC showed that (i) *TP53* was the most frequently inactivated gene (15/16; 93.7%) followed by *RB1* (5/16; 31.3%) and *KEAP1* (4/16; 25%), while *CRKL* and *MYB* genes were amplified in 4/16 (25%) cases and *MYC* in 3/16 (18.8%) cases and (ii) transcriptomic analysis identified two molecular subtypes including a Pure-LCC and an adenocarcinoma like-LCC (ADLike-LCC) characterized by different activated pathways and cell of origin. A schematic representation of the main findings of the present study is depicted in Fig. 4.

To date, only three studies reported a genomic characterization of marker-null LCC in 12 [4], 25 [1], and 7 cases [8], respectively. Karlsson et al*.* reported that 11/12 (91.7%) LCC had *TP53* mutations and 1/12 (8.3%) an activating mutation in *MET*, while none had *KRAS* or *RB1* alterations [4]. Chan et al*.* identified *TP53* mutations in 24/25 (96%) cases, while 4/25 (16%) showed mutations in each *KRAS* and *RB1* genes [1]. Liang et al*.* found *TP53* alteration in 4/7 (57.1%) cases and *RB1* and *KRAS* each in 3/7 (42.8%) cases. Our study confirmed *TP53* as a key driver of LCC, as well as the frequent involvement of *RB1*, and identified *KEAP1* alterations in 25% of cases. Moreover, we report for the first time the amplification of *CRKL* and *MYB* genes in 4/16 (25%) cases and the evaluation of TML that had a median value of 4.4 muts/Mb.

Our comparative expression analysis identified two LCC transcriptomic entities, Pure-LCC and ADLike-LCC, which respectively overlap with the marker-null LCC and the LCC-AC-like subtypes reported by the only gene expression study performed on 12 marker-null LCC [5]. Interestingly, the TML was significantly different between Pure-LCC and ADLike-LCC (median 5.1 *vs.* 1.4 muts/Mb; *p* = 0.04),and the amplification of *CRKL* and/or *MYB* was restricted to the Pure-LCC subtype, occurring in 45.5% (5/11) of cases.

Transcriptomic analysis of the lung marker-null LCC performed by Karlsson et al*.* highlighted that this group had an expression profile distinct from that of LCNEC and ADC [5], characterized by gene ontology processes such as DNA replication, cell division, and cellular response to stress and oxidation–reduction processes. Our study confirmed these observations defining the Pure-LCC as a molecular class distinct from LCNEC and ADC, characterized by a greater number of mitoses compared to ADLike-LCC and a series of biological processes related to DNA repair due to replication stress. Recently, these processes have been included in the “replication stress signature” previously described by Dreyer et al*.* [3] in pancreatic cancer and by Thomas et al*.* [21] in SCLC. Part of this signature is the *ATR* pathway which showed a highly enriched score in Pure-LCC, suggesting a central distinctive activity of this pathway, thus paving the way for innovative therapeutic perspectives in Pure-LCC including the use of Berzosertib, an *ATR* inhibitor, tested in combination with topotecan in patients affected by platinum-resistant SCLC [21].

In contrast, the transcriptomic analysis of ADLike-LCC showed a distinctive overexpression of *NAPSA* and Surfactant family genes, typical of adenocarcinomas, together with the exclusive overexpression of *AIM2*, *DKK1*, *S100A8*, and *SERPINB4*. *NAPSA* overexpression was associated with the immunopositivity of NapsinA in normal lung tissue within hyperplastic pneumocytes and intra-alveolar macrophages, as previously described [13]. In this respect, the GSEA showed that the ADLike-LCC group had the highest proportion of macrophages compared to ADC, LCNEC, and Pure-LCC groups, and the deconvolution analysis highlighted a strong leukocyte infiltrate which sets up a “hot tumour” profile in ADLike-LCC in contrast to a “cold tumour” profile of the Pure-LCC samples. The GSEA also showed a positive correlation with several pathways related to the inflammatory response, including the *AIM2* inflammasome complex. The *AIM2* gene has been described as a tumour suppressor in early studies [2] but in NSCLC it appears to promote tumour growth as an oncogene in an inflammasome-dependent way [25]. A recent study correlated the presence of the *AIM2* inflammasome complex signature with drug sensitivity to the compounds AICAR, AT-7519, bosutinib, DMOG, and Z-LLNLE-CHO [15], suggesting a potential therapy for these tumour types.

From a clinicopathological point of view, the two molecular subgroups showed significant differences regarding age at diagnosis (*p* = 0.023) and the number of mitoses observed (*p* = 0.009), both higher in Pure-LCCs. The higher mitotic count may suggest more aggressive behaviour of Pure-LCC, among which death events were also higher. However, the limited number of cases analysed does not allow definitive conclusions based on statistical evidence to be drawn.

Transcriptomic analysis also suggested a different cell of origin for the two LCC molecular subtypes: alveolar cell for ADLike-LCC and tuft cell for Pure-LCC. Indeed, GSEA showed that ADLike-LCC had an expression profile close to that of the alveolar epithelium, while Pure-LCC expression profile was similar to that of the tuft cell-like profile described by Yamada et al*.* and characterized by co-expression of *POU2F3* and *FOXI1* genes [24]. Of note, a recent study on the transcriptional mechanism of the tuft cell lineage identified a critical transcriptional complex composed of *POU2F3*, *OCA-T1*, and *OCA-T2*; these interactions may become an important target for pharmacological blockade in tuft cell-like carcinomas [23].

In conclusion, our study split the histological marker-null LCC category into two different transcriptomic entities, with *POU2F3*, *FOXI1*, and *AIM2* genes as differential expression markers that might be probed by immunohistochemistry for the differential diagnosis between Pure-LCC and ADLike-LCC. GSEA revealed a profile compatible with tuft cell-like origin for Pure-LCC and an alveolar cell origin for ADLike-LCC. Finally, the identification of several signatures linked to replication stress in Pure-LCC and inflammasome complex in ADLike-LCC could be useful for designing new potential therapeutic approaches for these subtypes.

### Supplementary Information

Below is the link to the electronic supplementary material.

Supplementary figure 1. (PNG 6.83 MB)

High resolution image (TIF 1.17 MB)

Supplementary figure 1. (PNG 7.71 MB)

High resolution image (TIF 3.22 MB)

Supplementary figure 1. (PNG 7.82 MB)

High resolution image (TIF 11.3 MB)

Supplementary file2 (XLSX 2613 KB)

Supplementary file3 (DOCX 46 KB)

## Data Availability

Data are available upon request.

## Abbreviations

*ChgA*: Chromogranin-A
*CNV*: Copy number variation
*FFPE*: Formalin-fixed paraffin-embedded
*HR*: Hazard ratio
*Ki-67*: Ki67 index
*NapA*: NapsinA
*OS*: Overall survival
*Syn*: Synaptophysin
*TTF-1*: Thyroid transcription factor 1
*WHO*: World Health Organization
