# Supplementary figures and images for: Characterization of two transcriptomic subtypes of marker-null large cell carcinoma of the lung suggests different origin and potential new therapeutic perspectives
Source: Virchows Arch. 2024 Jan 3;484(5):777–88. doi: 10.1007/s00428-023-03721-4 (PMC11106141; doi:10.1007/s00428-023-03721-4)

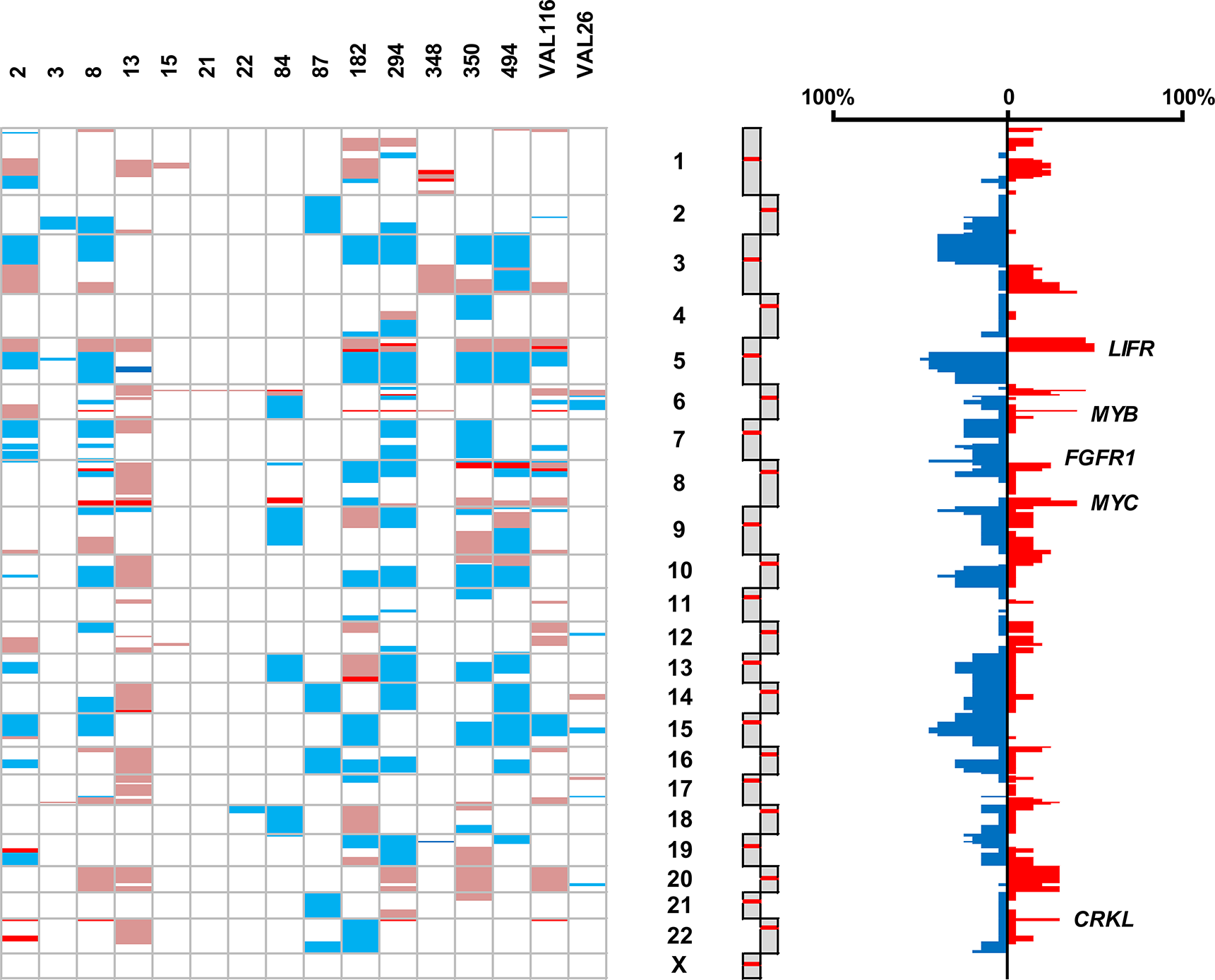

Supplement: Supplementary file 1 — (PNG 6.83 MB) [file 428_2023_3721_Fig5_ESM.png]

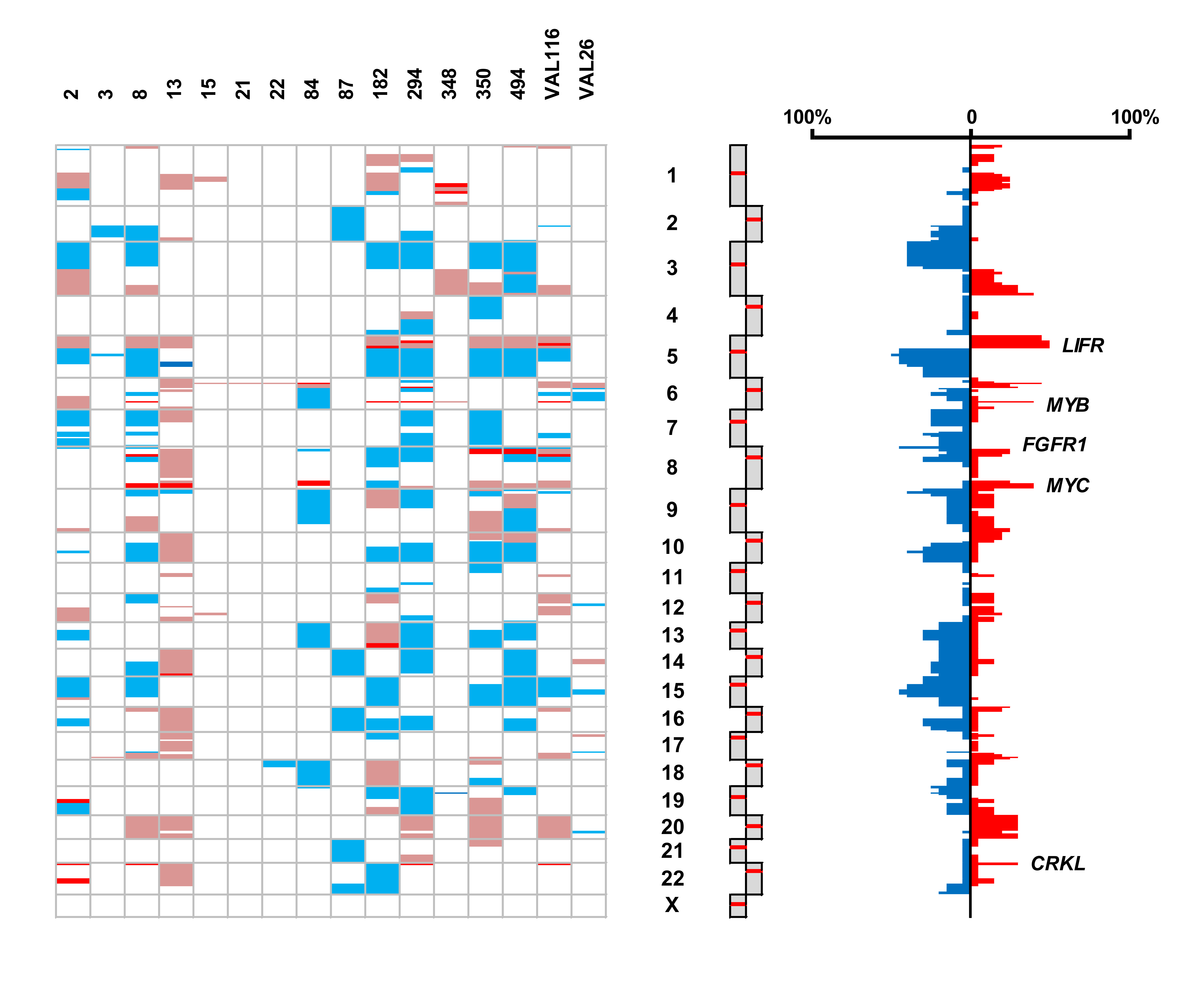

Supplement: Supplementary file 2 — High resolution image (TIF 1.17 MB) [file 428_2023_3721_MOESM1_ESM.tif]

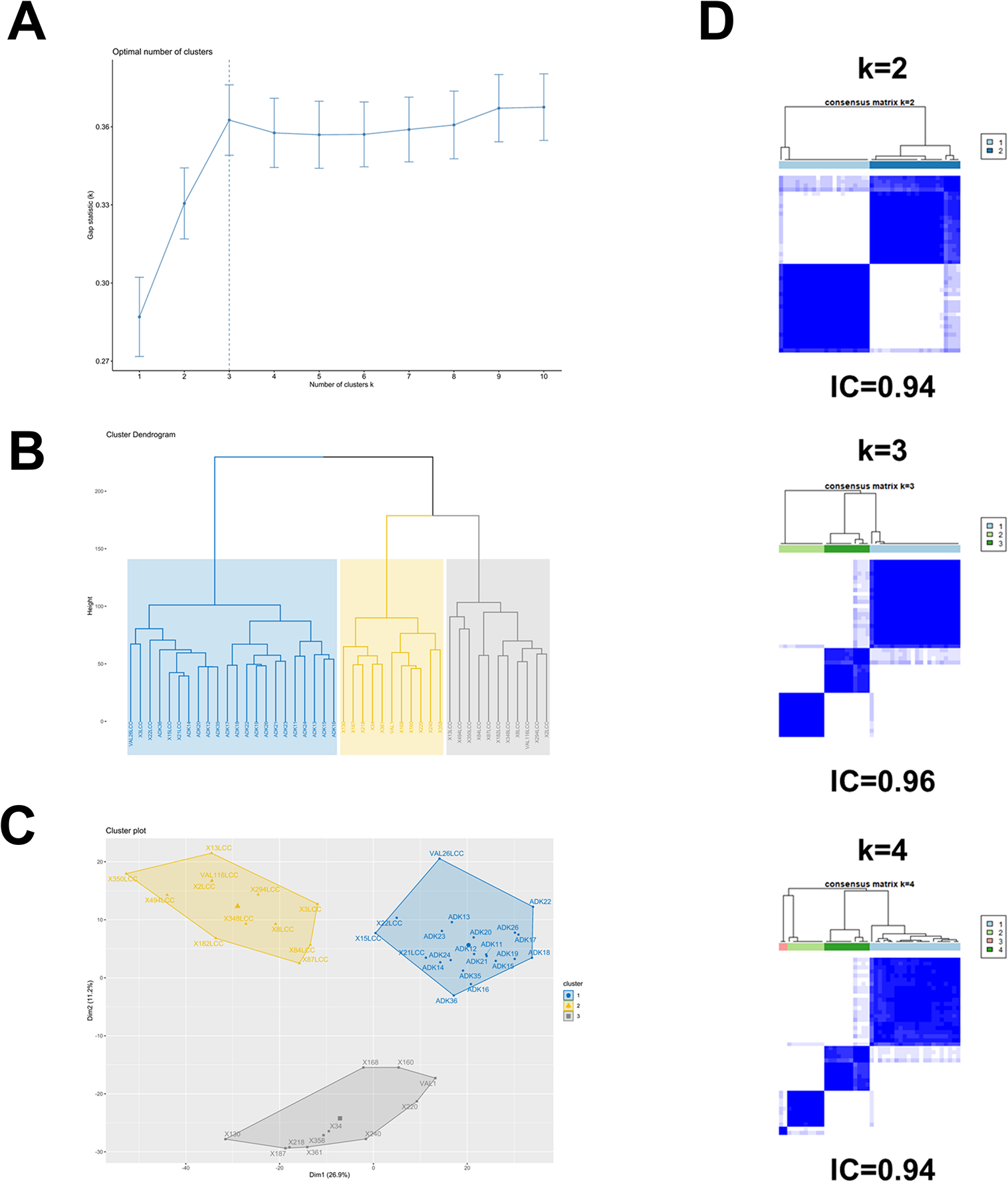

Supplement: Supplementary file 3 — (PNG 7.71 MB) [file 428_2023_3721_Fig6_ESM.png]

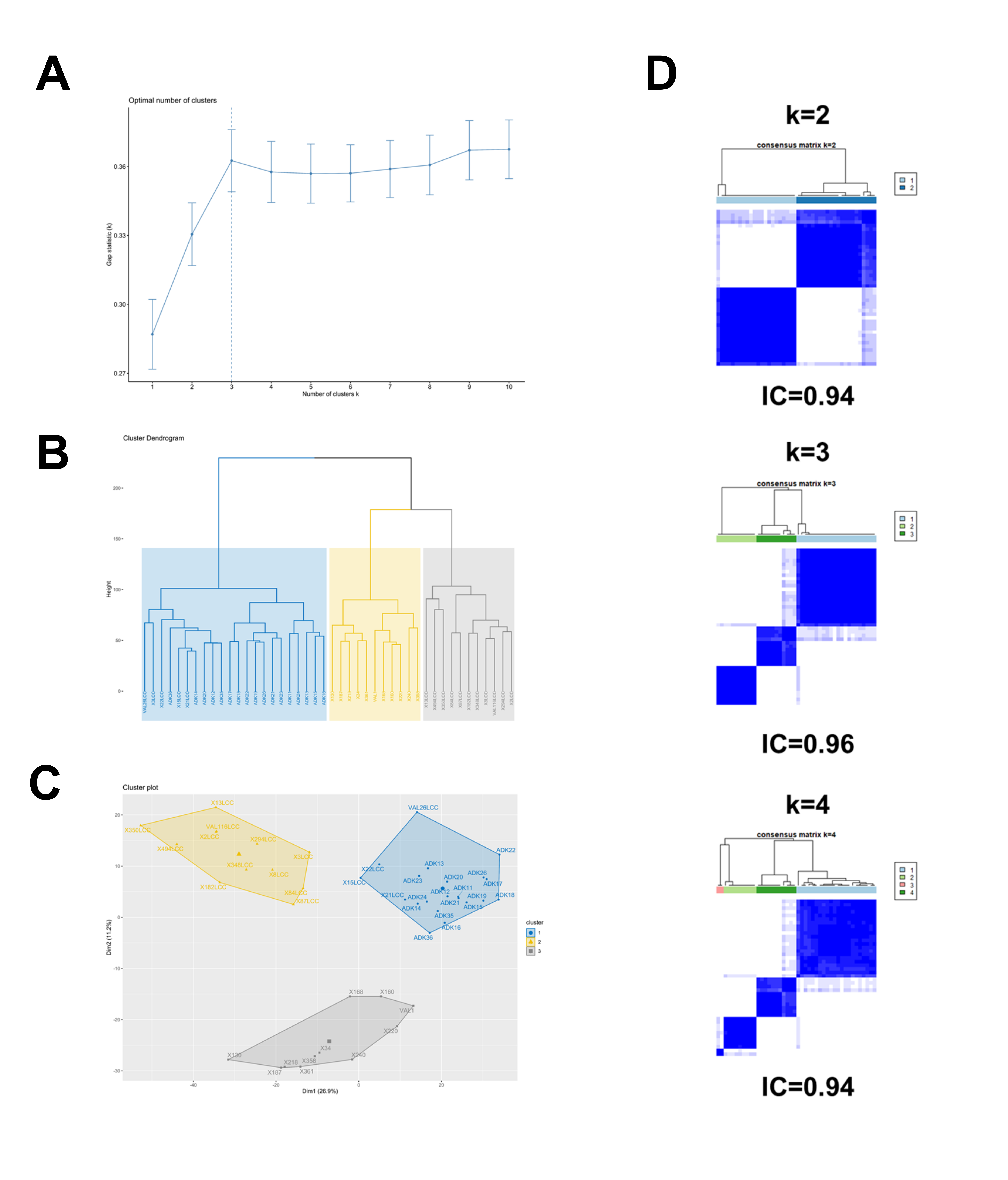

Supplement: Supplementary file 4 — High resolution image (TIF 3.22 MB) [file 428_2023_3721_MOESM2_ESM.tif]

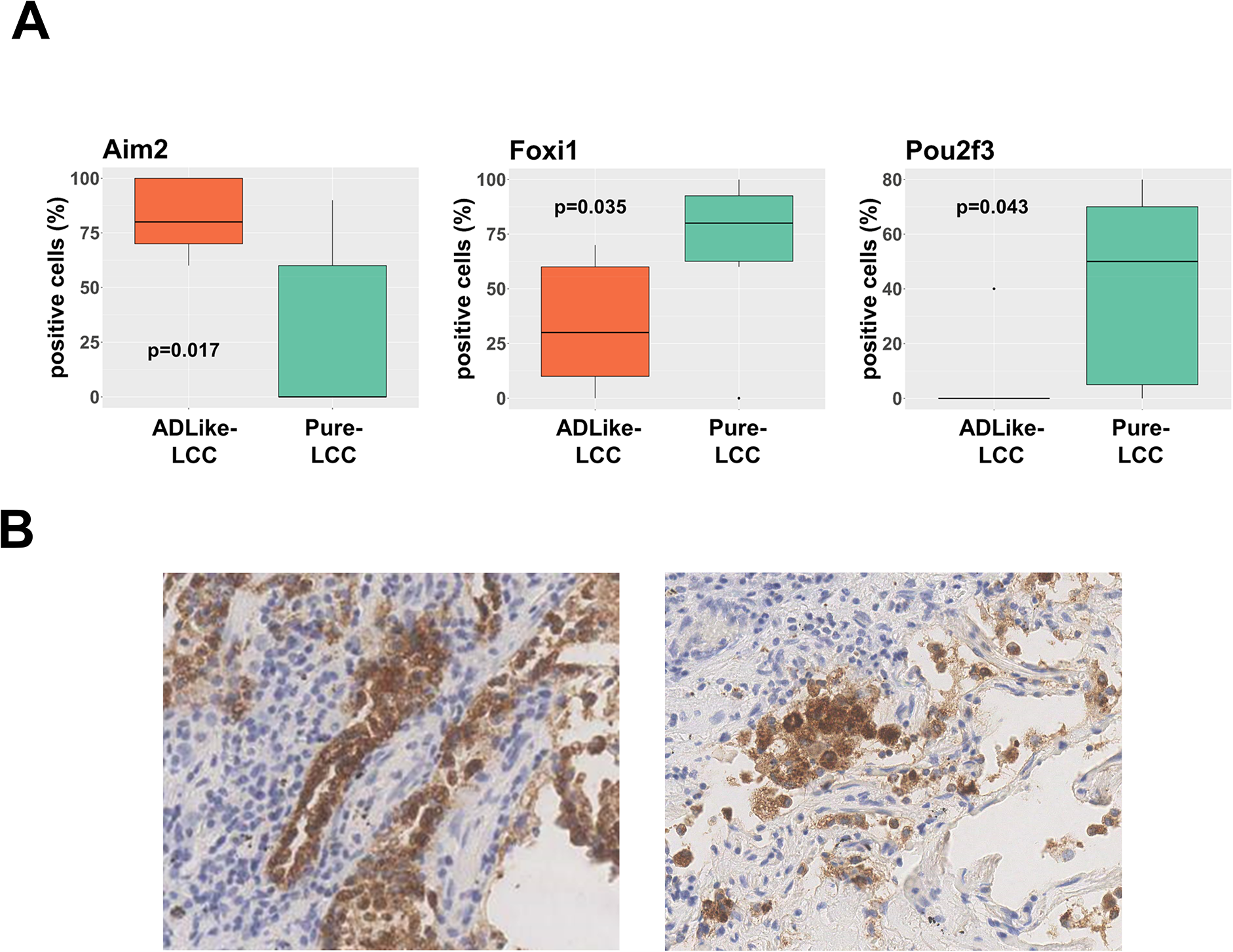

Supplement: Supplementary file 5 — (PNG 7.82 MB) [file 428_2023_3721_Fig7_ESM.png]

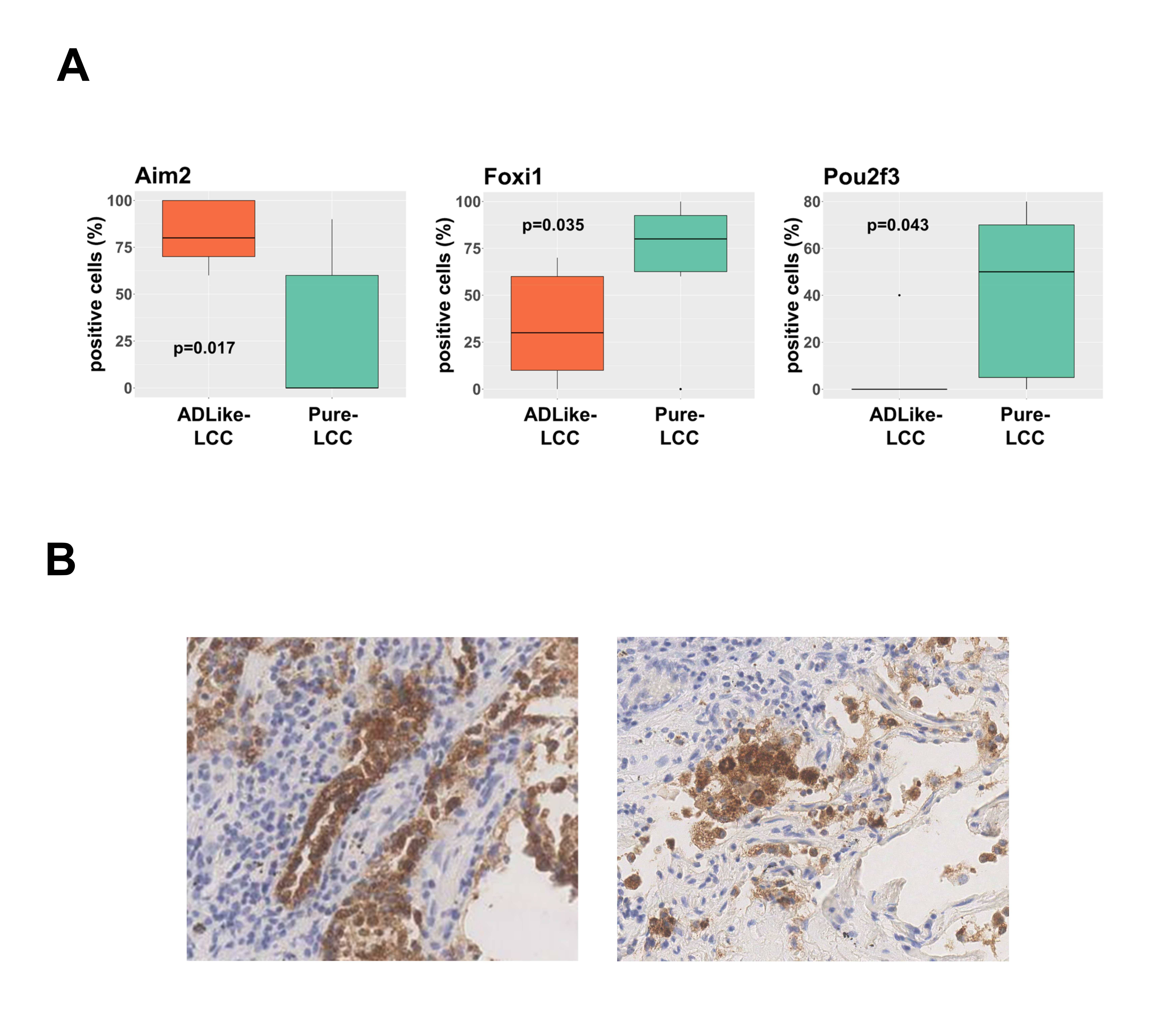

Supplement: Supplementary file 6 — High resolution image (TIF 11.3 MB) [file 428_2023_3721_MOESM3_ESM.tif]
