# Supplementary material for: Characterization of two transcriptomic subtypes of marker-null large cell carcinoma of the lung suggests different origin and potential new therapeutic perspectives
Source: Virchows Arch. 2024 Jan 3;484(5):777–88. doi: 10.1007/s00428-023-03721-4 (PMC11106141; doi:10.1007/s00428-023-03721-4)
Supplement: Supplementary file 8 — Supplementary file3 (DOCX 46 KB) [file 428_2023_3721_MOESM5_ESM.docx]

**Supplementary Methods**

*Mutational and copy number variation status of 409 cancer genes*

Sequencing was performed on Ion Torrent platform using 20 ng of DNA for each multiplex PCR amplification and subsequent library construction. The quality of the obtained libraries was evaluated by the Agilent 2100 Bioanalyzer on-chip electrophoresis (Agilent Technologies). Emulsion PCR was performed with the Ion Chef System (Thermo Fisher Scientific, MA, USA) to clonally amplify the libraries. Sequencing was run on the Ion S5XL (Thermo Fisher Scientific, MA, USA) loaded with Ion 540 Chip.

Data analysis, including alignment to the hg19 human reference genome and variant calling, was performed using Torrent Suite Software v.5.12 (Thermo Fisher Scientific, MA, USA). Filtered variants were annotated using a custom pipeline based on vcflib (https://github.com/ekg/vcflib), SnpSift[5], Variant Effect Predictor (VEP)[16] and NCBI RefSeq database. Additionally, alignments were visually verified with the Integrative Genomics Viewer (IGV) v2.8 [17] to further confirm the presence of identified mutations.

*Tumor mutational load and mutational signatures*

CNV was evaluated using OncoCNV v6.8[3]. BAM files obtained by the sequencing of tumor samples were compared to BAM files obtained from blood samples. The software includes a multi-factor normalization and annotation technique enabling the detection of large copy number changes from amplicon sequencing data and permits to visualize the output per chromosome. According to Krijgsman et al.[11], focal amplification was considered when copy number variation was limited in size to less than 3Mb and involved an oncogene. The gene was considered amplified when the number of copies exceeded 4.

TML is calculated using a specific algorithm of the Ion Reporter software (Thermo Fisher Scientific, MA, USA) and is expressed as the number of mutations per megabase (muts/Mb), where the number of mutations include nonsynonymous (missense and nonsense single nucleotide variants (SNVs), plus insertion and deletion variants (InDels) detected per megabase (Mb) of exonic sequences.

The signatures of somatic mutations (mutational spectrum) of individual tumors were obtained considering six major mutation classes: C>T (G:C>A:T); C>A (G:C>T:A); C>G (G:C>C:G); T>A (A:T>T:A); T>C (A:T>G:C); T>G (A:T>C:G) [1, 7]. Mutational Signatures in Cancer (MuSiCa) software [6] was used to obtain specific signatures for each sample. The software used vcf files to align them to the hg19 human reference genome using targeted sequencing parameters. The different types of base-pair substitutions, comprising all nonsynonymous missense and nonsense single nucleotide variants (SNVs), were normalized per megabase (Mb) of exonic sequence. The percentage of each group in each sample was computed.

*Fusion gene and splice variant detection by RT-PCR.*

The assay detects the main chromosomal translocations involving the *ALK*, *ROS1*, *RET* genes and the loss of exon 14 in the *MET* gene. Each oligonucleotide mixture allows the co-amplification of one or more fusions and of an endogenous control gene. A negative control and a positive control containing a mixture of synthetic RNA-DNA sequences corresponding to the major fusions detected by the kit was used in each run. To prepare the assay, 200 ng of RNA quantified by Qubit RNA HS Assay Kit (Thermo Fisher Scientific, MA, USA), was used in accordance with EasyPGX Protocol. Data analysis was done using AriaDx Analysis software v1.4 (Agilent Technologies) and EasyPGX Analysis Software using default parameters (Easy PGX platform, Diatech Pharmacogenetics, Jesi, Italy).

*Expression analysis by Next-Generation Sequencing.*

The Ampliseq Transcriptome Human Gene Expression Kit (Thermo Fisher Scientific) was used to analyze the expression status of 20,815 human genes. Libraries were prepared using AmpliSeq technology and 1 µg of retro-transcribed RNA for each multiplex PCR amplification. Clonal amplification was performed using the Ion Chef System (Thermo Fisher Scientific). Sequencing was run on the Ion S5XL (Thermo Fisher Scientific) loaded with Ion 540 Chip. The AmpliSeqRNA plugin was used to generate expression data (counts per transcript) for each sample. Counts were normalized and transformed using the "DESeq2” package for R and variance-stabilized read counts (vst), enabling the comparison of samples with different library sizes[13]. We then corrected the batch effect using ComBat, described in Johnson et al.[9]. Visualization and clustering were performed using the "ComplexHeatmap" package for R.[8]. The normalized and corrected dataset is used for a semi-supervised consensus clustering to unveil the similarities inside and between the histological classes. For this operation, we kept only the genes (HGVs) with the most variable expression (genes explaining 70% of the total variance) which accounted in 2109 HGVs.

The gap statistic method[19] was used on normalized counts of the HGVs to estimate the best number of clusters (k) which resulted to be k=3. Considering the HGVs and the best number of clusters estimated (k), the sample-to-sample distance between samples was computed using the hybrid hierarchical k-means approach. Principal component analysis (PCA) and hierarchical dendrogram was performed to show the relationships between samples[4]. To verify the resulting associations between samples, unsupervised consensus clustering was applied using ConsensusClusterPlus package of R[20]. A consensus matrix for the identified cluster solution was performed using the ConsensusClusterPlus package of R[20] with hierarchical clustering, applying average linkage and a Pearson correlation-based distance, while hybrid hierarchical k-means was used to cluster the samples.[2] Cluster-consensus (CLC), the average pairwise item consensus (IC) of items in a consensus cluster, are reported below for each solution.

A pan-lung map was obtained using the Uniform Manifold Approximation and Projection (UMAP) method on the genes with the previously selected genes (n=2109). UMAP is a dimensionality reduction method based on manifold learning techniques, which are adapted to non-linear data in contrast with the commonly used principal component analysis (PCA) method. First, it builds a topological representation of the high-dimensional data, and second, it finds the best low-dimensional representation of this topological structure[15]. UMAP representations were generated using the umap function from the R package umap (v. 0.2.5.0)[10]. All the parameters were set to their default values except then neighbors’ parameter. This parameter defines the number of neighbors considered to learn the structure of the topological space. Varying this parameter from small to large values enables the user to find a trade-off between local and global preservation of the space, respectively. We built the pan-lung map by setting the n_neighbors parameter to 65, which corresponds to the total number of samples.

We computed differential expressed genes following limma workflow. We intersected the results of all the pairwise comparisons and determined how much a gene was frequently appearing as DE for a specific cluster against every other and poorly appearing in the comparisons not including the group.

Next, we analysed the samples at pathway-level. We downloaded gene sets from MSigDB[12, 18] and determined the cluster-specific enriched gene sets using the normalized and batch-corrected count matrix. We applied GSEA using the GAGE R package[14] between clusters to get pairwise significant up and down regulated pathways. We used an approach based on the ssGSEA score[18] for determining the biological processes differently enriched between all the clusters. The ssGSEA score determines how much the genes in a particular set are co-ordinately up- or down-regulated within a specific sample. We assessed the ssGSEA score for each pair of sample and gene set. We represented each pathway in a cluster with the mean score obtained by its members. We performed a z-score normalization of the pathway scores in the clusters. We ranked the biological processes for each cluster based on the Euclidian distance between its enrichment score and the mean of the other groups. We then selected the top pathways in a rank list to characterize the clusters.

**References**

1. Alexandrov LB, Nik-Zainal S, Wedge DC, Aparicio SA, Behjati S, Biankin AV, Bignell GR, Bolli N, Borg A, Borresen-Dale AL, Boyault S, Burkhardt B, Butler AP, Caldas C, Davies HR, Desmedt C, Eils R, Eyfjord JE, Foekens JA, Greaves M, Hosoda F, Hutter B, Ilicic T, Imbeaud S, Imielinski M, Jager N, Jones DT, Jones D, Knappskog S, Kool M, Lakhani SR, Lopez-Otin C, Martin S, Munshi NC, Nakamura H, Northcott PA, Pajic M, Papaemmanuil E, Paradiso A, Pearson JV, Puente XS, Raine K, Ramakrishna M, Richardson AL, Richter J, Rosenstiel P, Schlesner M, Schumacher TN, Span PN, Teague JW, Totoki Y, Tutt AN, Valdes-Mas R, van Buuren MM, van 't Veer L, Vincent-Salomon A, Waddell N, Yates LR, Australian Pancreatic Cancer Genome I, Consortium IBC, Consortium IM-S, PedBrain I, Zucman-Rossi J, Futreal PA, McDermott U, Lichter P, Meyerson M, Grimmond SM, Siebert R, Campo E, Shibata T, Pfister SM, Campbell PJ, Stratton MR (2013) Signatures of mutational processes in human cancer Nature 500:415-421. doi: 10.1038/nature12477

2. Bernard C, Harrison R, Yi P, Phang CT (2005) Novel Hybrid Hierarchical-K-means Clustering Method (H-K-means) for Microarray Analysis2005 IEEE Computational Systems Bioinformatics Conference - Workshops (CSBW'05). IEEE Computer Society, pp. 105-108

3. Boeva V, Popova T, Lienard M, Toffoli S, Kamal M, Le Tourneau C, Gentien D, Servant N, Gestraud P, Rio Frio T, Hupe P, Barillot E, Laes JF (2014) Multi-factor data normalization enables the detection of copy number aberrations in amplicon sequencing data Bioinformatics 30:3443-3450. doi: 10.1093/bioinformatics/btu436

4. Chen B, Harrison R, Pan Y, Tai PC (2005) Novel Hybrid Hierarchical-K-means Clustering Method (H-K-means) for Microarray AnalysisProceedings of the 2005 IEEE Computational Systems Bioinformatics Conference - Workshops. IEEE Computer Society, pp. 105–108

5. Cingolani P, Patel VM, Coon M, Nguyen T, Land SJ, Ruden DM, Lu X (2012) Using Drosophila melanogaster as a Model for Genotoxic Chemical Mutational Studies with a New Program, SnpSift Frontiers in genetics 3:35. doi: 10.3389/fgene.2012.00035

6. Diaz-Gay M, Vila-Casadesus M, Franch-Exposito S, Hernandez-Illan E, Lozano JJ, Castellvi-Bel S (2018) Mutational Signatures in Cancer (MuSiCa): a web application to implement mutational signatures analysis in cancer samples BMC Bioinformatics 19:224. doi: 10.1186/s12859-018-2234-y

7. Erson-Omay EZ, Caglayan AO, Schultz N, Weinhold N, Omay SB, Ozduman K, Koksal Y, Li J, Serin Harmanci A, Clark V, Carrion-Grant G, Baranoski J, Caglar C, Barak T, Coskun S, Baran B, Kose D, Sun J, Bakircioglu M, Moliterno Gunel J, Pamir MN, Mishra-Gorur K, Bilguvar K, Yasuno K, Vortmeyer A, Huttner AJ, Sander C, Gunel M (2015) Somatic POLE mutations cause an ultramutated giant cell high-grade glioma subtype with better prognosis Neuro Oncol 17:1356-1364. doi: 10.1093/neuonc/nov027

8. Gu Z, Eils R, Schlesner M (2016) Complex heatmaps reveal patterns and correlations in multidimensional genomic data Bioinformatics 32:2847-2849. doi: 10.1093/bioinformatics/btw313

9. Johnson WE LCaRA (2007) Adjusting batch effects in microarray expression data using empirical Bayes methods. Biostatistics

10. Konopka T (2019) umap: Uniform Manifold Approximation and Projection.

11. Krijgsman O, Carvalho B, Meijer GA, Steenbergen RD, Ylstra B (2014) Focal chromosomal copy number aberrations in cancer-Needles in a genome haystack Biochim Biophys Acta 1843:2698-2704. doi: 10.1016/j.bbamcr.2014.08.001

12. Liberzon A SA, Pinchback R, Thorvaldsdóttir H, Tamayo P, Mesirov JP (2011) Molecular signatures database (MSigDB) 3.0 Bioinformatics

13. Love MI, Huber W, Anders S (2014) Moderated estimation of fold change and dispersion for RNA-seq data with DESeq2 Genome Biol 15:550. doi: 10.1186/s13059-014-0550-8

14. Luo W FM, Shedden K, Hankenson KD, Woolf PJ. (2009) GAGE: generally applicable gene set enrichment for pathway analysis. BMC Bioinformatics

15. McInnes LH, J.; Melville, J. (2018) UMAP: Uniform Manifold Approximation and Projection for Dimension Reduction. doi: arXiv:1802.03426

16. McLaren W, Pritchard B, Rios D, Chen Y, Flicek P, Cunningham F (2010) Deriving the consequences of genomic variants with the Ensembl API and SNP Effect Predictor Bioinformatics 26:2069-2070. doi: 10.1093/bioinformatics/btq330

17. Robinson JT, Thorvaldsdottir H, Winckler W, Guttman M, Lander ES, Getz G, Mesirov JP (2011) Integrative genomics viewer Nature biotechnology 29:24-26. doi: 10.1038/nbt.1754

18. Subramanian A TP, Mootha VK, et al. (2005) Gene set enrichment analysis: a knowledge-based approach for interpreting genome-wide expression profiles. Proc Natl Acad Sci USA

19. Tibshirani R, Walther G, Hastie T (2001) Estimating the number of clusters in a data set via the gap statistic Journal of the Royal Statistical Society: Series B (Statistical Methodology) 63:411-423. doi: 10.1111/1467-9868.00293

20. Wilkerson MD, Hayes DN (2010) ConsensusClusterPlus: a class discovery tool with confidence assessments and item tracking Bioinformatics 26:1572-1573. doi: 10.1093/bioinformatics/btq170
